# Neoadjuvant Immunotherapy Improves Treatment for Early Resectable Non-Small-Cell Lung Cancer: A Systematic Review and Meta-analysis

**DOI:** 10.1155/2022/2085267

**Published:** 2022-09-30

**Authors:** Peng Dong, Yu Yan, Liyuan Yang, Danzhu Wu, Hui Wang, Yajuan Lv, Jiandong Zhang, Xinshuang Yu

**Affiliations:** ^1^Department of Oncology, The First Affiliated Hospital of Shandong First Medical University and Shandong Provincial Qianfoshan Hospital, Shandong Key Laboratory of Rheumatic Disease and Translational Medicine, Shandong Lung Cancer Institute, Taian, Shandong, China; ^2^Clinical Medical College of Jining Medical University, Jining, Shandong Province, China

## Abstract

**Objective:**

Immunotherapy has shown better efficacy and less toxicity than chemotherapy in the treatment of non-small-cell lung cancer (NSCLC) at advanced stage. This study evaluates the safety and efficacy of neoadjuvant immunotherapy for resectable NSCLC.

**Methods:**

Literature examination was performed by searching the PubMed, the Cochrane Library, and Embase for articles evaluating the efficacy and safety of neoadjuvant immunotherapy for resectable NSCLC. The 95% confidence interval (CI) and effect sizes (ES) were evaluated. Heterogeneity and subgroup analysis were performed. Meta-analysis was carried out using Stata BE17 software.

**Results:**

In total, 678 patients from eighteen studies were recruited in this meta-analysis. The pathological complete response (pCR) and major pathological response (MPR) were used to evaluate the efficacy of neoadjuvant immunotherapy. Significantly higher MPR values were observed in neoadjuvant immunotherapy (MPR : ES = 0.44; 95% CI: 0.33–0.55; pCR : ES = 0.22; 95% CI: 0.15–0.30) compared with neoadjuvant chemotherapy (MPR < 25% and PCR : ES = 2%–15%). Treatment-related adverse events (TRAE), surgical resection rate, surgical delay rate, and incidence of surgical complications were used to evaluate the safety. In summary, ES values for the incidence of TRAE, incidence of surgical complications, and surgical delay rate were 0.4, 0.24, and 0.04, respectively, that were significantly lower than those for neoadjuvant chemotherapy (95% CI: 0.04–0.90; 0.22–0.75; and 0.01–0.10, respectively). The mean surgical resection rate of 89% was similar to the reported 75%–90% resection rate with neoadjuvant chemotherapy (OR = 7.61, 95% CI: 4.90–11.81).

**Conclusion:**

Neoadjuvant immunotherapy is safe and effective for resectable NSCLC.

## 1. Introduction

Lung cancer ranks the first in its incidence and mortality worldwide. Based on the global cancer statistics report in 2021, the mortality of lung cancer accounts for 18% of all cancer-related deaths. NSCLC is the most common histopathological type of lung cancer, accounting for about 80% of cases [1]. Surgical resection is the treatment of choice for patients with NSCLC at early stage, but only 25% of patients are eligible for surgery, and these patients remain at high risk for recurrence and death after surgery. It was reported that neoadjuvant therapy improved the rate of surgical resection. However, whether neoadjuvant therapy could improve the survival of patients with resectable NSCLC remains controversial [2]. A systematic review on 32 randomized clinical trials that involved 10000 patients showed no significant difference in survival between patients treated with preoperative neoadjuvant and postoperative adjuvant chemotherapy [3]. The pCR rate for neoadjuvant chemotherapy is 2% to 15%, and concurrent neoadjuvant radiotherapy does not significantly improve pCR in patients, and there is a high incidence of toxicity with the combination of radiotherapy and chemotherapy, with grade 3 or 4 events occurring in 45% to 60% of patients in the chemoradiotherapy combination group [4].

Over the past few years, the application of immune checkpoint inhibitors (ICIs) has greatly changed the treatment modalities of lung cancer. ICIs used in clinics mainly include programmed death-1 (PD-1) and its ligand (PD-L1) inhibitor, and cytotoxic T-lymphocyteantigen-4 (CTLA-4) inhibitor. Inhibition of these regulatory pathways can activate immune T-cell responses against tumors [5, 6]. Currently, most clinical trials on neoadjuvant immunotherapy, such as CheckMate 816, IMpower030, NEOSTAR, LCMC3, and NEOMUN, for resectable NSCLC are still ongoing. Among these trials, some data have been presented at the European Society of Medical Oncology (ESMO), American Society of Clinical Oncology (ASCO), and other international meetings. Preliminary results suggest that neoadjuvant immunotherapy is efficient and improves survival in patients with resectable NSCLC [7]. Therefore, a meta-analysis on neoadjuvant immunotherapy clinical trials with published results will provide options for neoadjuvant therapy and guidance for future clinical trials.

Based on the existing data, this meta-analysis aims to demonstrate the safety and effectiveness of neoadjuvant immunotherapy for resectable NSCLC. The results of this analysis will provide guidance for the comprehensive treatment strategy for NSCLC.

## 2. Materials and Methods

### 2.1. Strategy for Literature Search

A computerized search on PubMed, EMBASE, Cochrane, and ClinicalTrials databases was used to search articles reporting on the evaluation of the efficacy of neoadjuvant immunotherapy for resectable NSCLC, and for the most recent data on a subset of ongoing clinical trials of neoadjuvant immunotherapy for NSCLC at international oncology conferences such as ASCO and ESMO. The search keywords included “NSCLC” and Mesh words such as “non-small-cell lung carcinoma,” “non-small-cell lung cancer,” “resectable,” “resectability,” “neoadjuvant,” and “immunotherapy.” To increase the rate of positive literature searches by increasing the search limit “title/Abstract.” References from all articles related to this meta-analysis were manually screened by reading the title, abstract, or full text to collect articles as comprehensive as possible and avoid missing articles. The articles were collated and categorized by two investigators using Endnote X9 software independently. References from relevant conference reports were also reviewed manually.

### 2.2. The Criteria of Inclusion and Exclusion

The criteria of inclusion were as follows: (I) patients with stage I-III resectable NSCLC who had a clear pathological and imaging diagnosis; (II) ICIs are used in registered clinical trials or clinical practice; (III) the inclusion of complete or at least one of the main study endpoints, such as pCR, MPR, treatment-related adverse event rate (TRAE), surgical resection rate, surgical complication rate, surgical delay rate, and others. Exclusion criteria were as follows: (I) patients with NSCLC present with local progression or develop tumor metastasis that precludes surgery; (II) previous treatment with any ICIs; (III) the primary study focus was not on rates of MPR, pCR, TRAE, surgical complications, surgical resection, or delayed surgery; (IV) fewer than 10 patients included in the study; (V) there are no valid data to evaluate the safety and efficacy of neoadjuvant immunotherapy; and (VI) violation of any one of the above inclusion criteria.

### 2.3. Data Abstracted

The literature was screened by two investigators independently based on the established inclusion and exclusion criteria. The investigators reviewed the literature by establishing group management classification for inclusion and exclusion, and removed duplicates. Each article was evaluated multiple times to ensure data integrity and accuracy. The data extraction contents include published year, first author, clinical trial, study type, NCT number, type of article, study phase, ICI, dose of ICI, main inclusion criteria, median age, proportion of gender, estimated enrollment, and sample size. The primary and secondary outcome endpoints are MPR, pCR, incidence of TRAE, surgical resection rate, incidence of surgical complications, and surgical delay rate. In case of controversy over inclusion in the literature by the two investigators, adjudication by a third investigator was considered.

### 2.4. Statistical Analysis

Stata BE17 software was used for meta-analysis. Because the included studies are mostly single-arm clinical trials, the pCR and MPR are used the main effect indicators. The 95% CI and ES are effect measures. The *χ*^2^ test and *I*^2^ test were used to determine heterogeneity. If the heterogeneity is significant, the random effect model is used; otherwise, the fixed-effect model is used. *P* < 0.05 was considered statistically significant. Subgroup analysis was used to explore the source of heterogeneity.

### 2.5. Assessment of Study Quality and Publication Bias

The quality of the studies was assessed by the recommended risk of bias assessment tool in the Cochrane Handbook 5.1.0. The assessment included the following: (1) allocation concealment; (2) random allocation method; (3) whether the outcome was assessed by a blind method; (4) whether to adopt a blind method for the participants and researchers; (5) selective reporting of outcomes; (6) completeness of outcome data; and (7) other bias. The literature quality was evaluated by two researchers independently. If there is a dispute between them, the decision of a third researcher is considered.

## 3. Results

### 3.1. Literature Search Results

**Figure 1:**
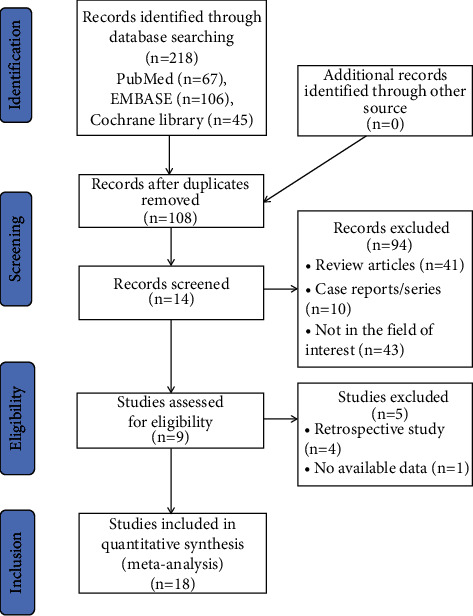
Results of literature search.

Based on the research strategy, 218 studies were recruited from the first search, and 110 duplicate articles were excluded. Additionally, 81 were eliminated based on their titles and abstracts. Finally, 29 articles were selected for comprehensive and detailed examination. After reading the full text carefully, ten studies were removed because they did not meet the criteria of inclusion. Ultimately, eighteen articles, including a total of 678 patients, were used for the analysis. The details of all included studies are shown in Table 1. Among the eighteen included studies, two are dual-armopen-label randomized controlled clinical trials and sixteen are single-armopen-label cohort studies. The selection process of the study is shown in Figure 1.

### 3.2. The Primary Outcome

#### 3.2.1. The Efficacy of Neoadjuvant Immunotherapy

**Figure 2:**
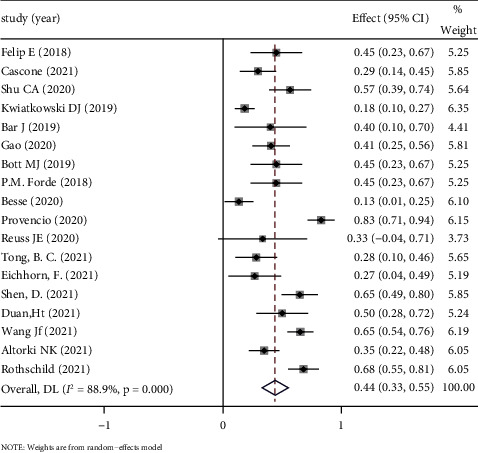
Forest map of MPR.

*(1) MPR*. MPR is defined by less than 10% residual viable tumor cells in the resected primary tumors [26]. The mean MPR was 45.3%. The test result of heterogeneity was *I*^2^ = 88.9% (>50%) and *P* < 0.1 in *Q* test. These results suggested that there is a high degree of heterogeneity in this study. Therefore, random effect was selected for meta-analysis. Based on the meta-analysis of random effects, the effect amount summarized in 18 studies is 0.44 (95% CI: 0.33∼0.55, *P* < 0.05), suggesting that neoadjuvant immunotherapy is effective for MPR of resectable NSCLC, and the effective rate is 0.44. See Figure 2 forest map for details.

**Figure 3:**
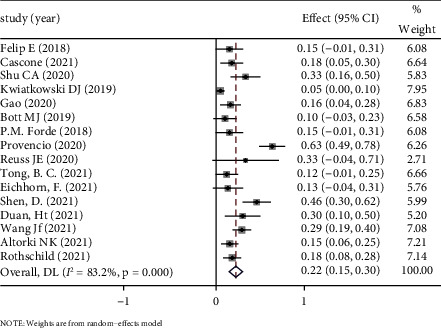
Forest map of pCR.

*(2) pCR*. The definition of pCR is the absence of viable tumor cells in the resected primary tumors. pCR is another powerful indicator for predicting the efficacy of neoadjuvant therapy [27]. The combined ES was 0.22 (95% CI: 0.15–0.30) with a statistically significant difference (*P* < 0.05) and, as a whole, was in favor of neoadjuvant immunotherapy. A random-effect model was used because significant heterogeneity in the 17 studies was found (*P* < 0.1, *I*^2^ = 83.2%). The average rate of pCR of 17 eligible studies was 21.64%. See Figure 3 forest map for details.

#### 3.2.2. The Safety of Neoadjuvant Immunotherapy

**Figure 4:**
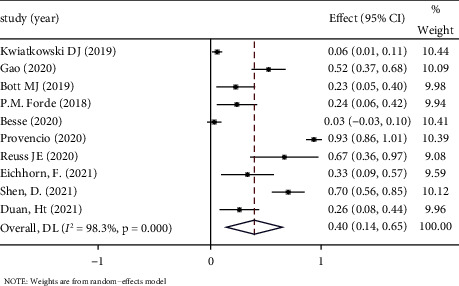
Forest map of the incidence of TRAE.

*(1) The Incidence of TRAE*. TRAE is a key metric for assessing the safety of neoadjuvant immunotherapy and refers to adverse events that occur as a result of using ICIs. The incidence of TRAE is evaluated by the National Cancer Institute Common Terminology Criteria for adverse events (NCICTCAE), version 4. Among the included clinical studies, only 10 described the incidence of TRAE in 344 patients. The mean incidence was 36.05%, and the combined ES was 0.40 (95% CI: 0.14–0.65), *P* < 0.05. The random-effect model was applied for analysis because of significant heterogeneity (*P* < 0.1, *I*^2^ = 98.3%). See Figure 4 forest map for details.

**Figure 5:**
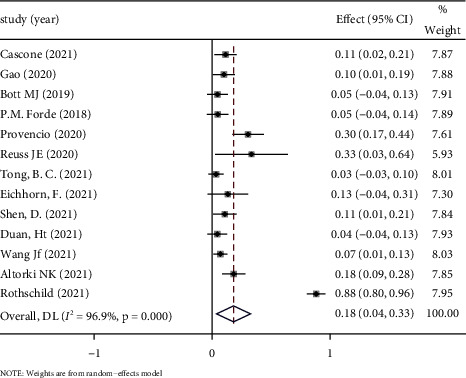
Forest map of grade 3 or higher TRAEs.

*(2) Grade 3 or Higher TRAEs*. The heterogeneity test indicated *I*^2^ = 96.9%, which is >50%, and *P* < 0.1 in *Q* test. This result suggested that there is a significantly high degree of heterogeneity of the study. Random effect was selected for meta-analysis. Based on the meta-analysis of random effects, the effect amount summarized by 13 studies is 0.18, and the 95% CI is 0.04∼0.33. There was one patient died due to bronchopleural fistula following steroid therapy for pneumonia [9], and others were mostly manageable adverse events such as pneumonia, hypoxia, hyperoxia, and diarrhea, which did not lead to serious adverse outcomes or resulted in high postoperative mortality. See Figure 5 forest map for details.

**Figure 6:**
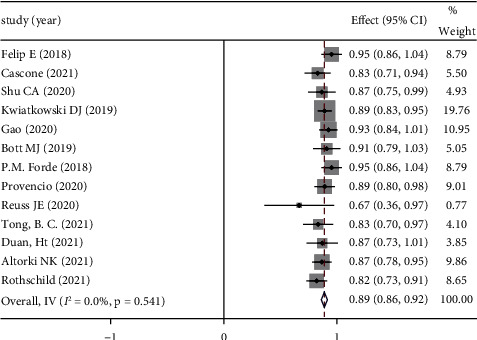
Forest map of surgical resection rate.

*(3) Surgical Resection Rate*. The surgical resection rate refers to the ratio of patients who undergo surgical resection to those who are expected to undergo surgery. The surgical resection rate is also an important indicator of safety in using neoadjuvant immunotherapy. According to the 18 included studies, the mean surgical resection rate was 90.37%. The combined ES was 0.89 (95% CI: 0.86–0.92), with insignificant heterogeneity. Therefore, the fixed-effect model was adopted for analysis (*P*=0.54, *I*^2^ = 0%). See Figure 6 forest map for details.

**Figure 7:**
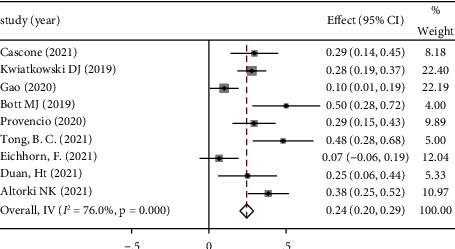
Forest map of incidence of surgical complication.

*(4) Incidence of Surgical Complication*. The incidence of surgical complication is commonly used to evaluate the safety of neoadjuvant immunotherapy. It refers to the procedure-related complications that occur perioperatively. The rates of surgical complications were provided in 9 studies. Because of the significant heterogeneity (*P* < 0.10, *I*^2^ = 76%), the random-effect model was adopted for analysis. The ES of these 9 studies was 0.24 (95% CI: 0.20–0.29). Among these studies, the average incidence of surgical complication was 28.53%, and the complication included atrial arrhythmia, bronchopleural fistulas, and air leaks. The prognosis of most of the complications was good. See Figure 7 forest map for details.

**Figure 8:**
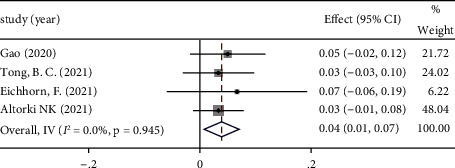
Forest map of surgical delay rate.

*(5) Surgical Delay Rate*. The surgery delay rate is defined as the ratio of patients who delay their surgery because of adverse events caused by neoadjuvant immunotherapy to all patients who are expected to having surgery. The surgery delay rate is commonly used to assess the safety of neoadjuvant immunotherapy. Data of surgical delay rate were obtained from 14 included studies. The ES of these 14 studies was 0.04 (95% CI: 0.01∼0.07). Because there was no remarkable heterogeneity found in these studies, the fixed-effect model was applied for analysis (*P*=0.945; *I*^2^ = 0%). See Figure 8 forest map for details.

### 3.3. Exploratory Subgroup Analysis

#### 3.3.1. Subgroup Analysis of ICIs Species

**Figure 9:**
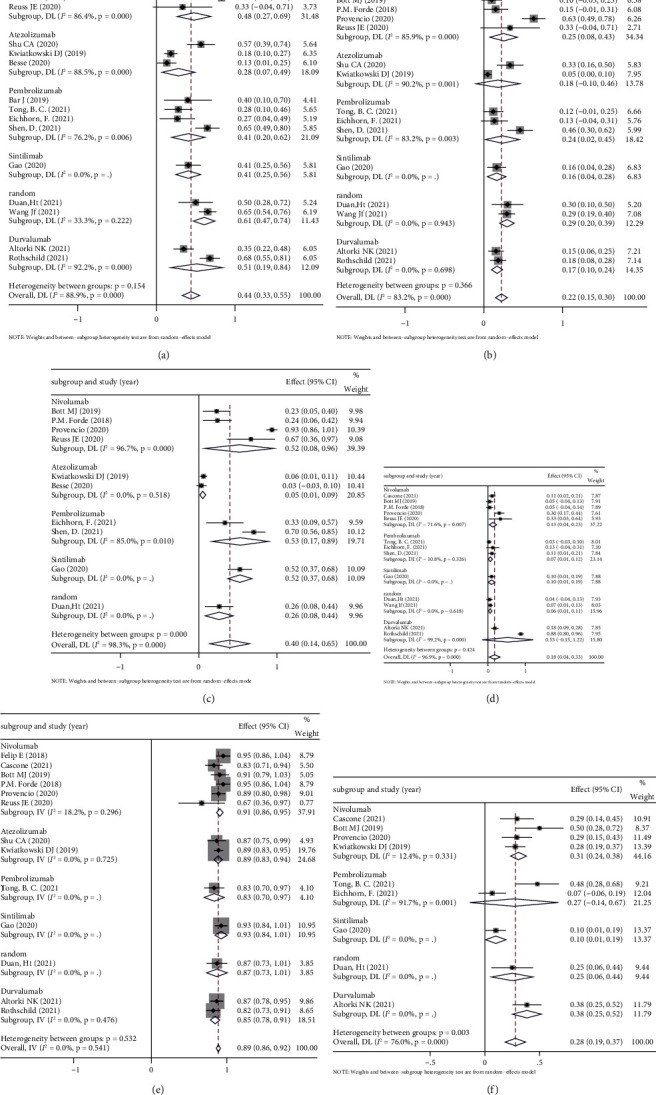
Subgroup analysis based on the type for (a) MPR, (b) pCR, (c) the incidence of TRAE, (d) grade 3 or higher TRAEs, (e) surgical resection rate, and (f) incidence of surgical complication.

To understand the association between the type of ICIs and the outcome of neoadjuvant immunotherapy, we performed subgroup analysis in 18 eligible studies, including four studies where patients were treated with pembrolizumab, three with atezolizumab, one with sintilimab, six with nivolumab, two with durvalumab, and two random ICIs. No significant findings were observed on the relationship in the type of ICIs about the safety and the efficacy of neoadjuvant immunotherapy, suggesting that no single ICIs have an absolute advantage in neoadjuvant immunotherapy. The details are shown in Figure 9.

#### 3.3.2. Subgroup Analysis of Treatment Mode

**Figure 10:**
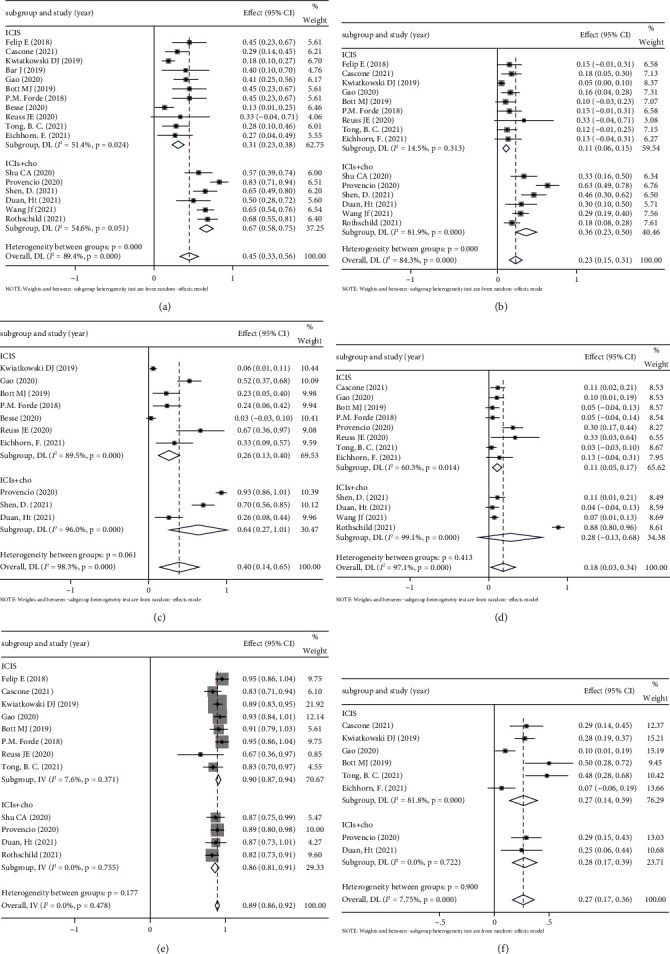
Subgroup analysis based on type for (a) MPR, (b) pCR, (c) the incidence of TRAE, (d) grade 3 or higher TRAEs, (e) surgical resection rate, and (f) incidence of surgical complication.

It is worth noting that not all the 18 eligible studies administered immunotherapy alone to patients. Among these studies, patients were treated with a combination of immunotherapy and chemotherapy in 6 studies. Besides, one study was radiotherapy combined with immunotherapy and subgroup analysis could not be performed. Because chemotherapy may augment the effects of immunotherapy by increasing antigen presentation, we performed subgroup analysis using treatment mode as a variable to exclude the effect of chemotherapy on the results of this meta-analysis. The results showed that neoadjuvant immunotherapy combined with chemotherapy was more effective in MPR and pCR, but the combination chemotherapy had more adverse effects, which did not significantly affect the safety of surgery. These results supported the safety and efficacy of neoadjuvant immunotherapy (Figure 10).

## 4. Discussion

Although there is controversial on the effect of neoadjuvant immunotherapy, the result of the current study demonstrates the safety and efficacy of using neoadjuvant immunotherapy for resectable NSCLC. Jia et al. [28] published a meta-analysis on neoadjuvant immunotherapy for resectable NSCLC, and we reached different conclusions by further searching the literature and performing the analysis. In this meta-analysis, the mean pCR of neoadjuvant immunotherapy was 21.64%, and the average MPR was 45.3% in the 18 included clinical studies. The efficacy of neoadjuvant immunotherapy in patients with resectable NSCLC is significantly higher than that of neoadjuvant chemotherapy, which has an MPR of less than 25% [29] and a pCR of approximately 2%–15% [30, 31, 32]. Our results confirmed the safety of using neoadjuvant immunotherapy. In this meta-analysis, TRAE occurred at an average rate of 36.0%, which was superior to neoadjuvant chemotherapy with a 40% toxicity [33, 34]. Moreover, the mean surgical resection rate was 90.37%, implying that the use of preoperative neoadjuvant immunotherapy did not decrease the surgical resection rate of patients compared with the 75–90% [34, 35] surgical resection rate reported by neoadjuvant chemotherapy. After subgroup analysis, it was not seen that the efficacy of one ICI was significantly better than the other. However, due to the small data size and excessive heterogeneity, this result needs further confirmation. However, based on the subgroup analysis on neoadjuvant immunotherapy alone and neoadjuvant immunotherapy in combination with neoadjuvant chemotherapy, we believe that the combination of the two can achieve better MPR and pCR, although the combination therapy has more adverse effects and has little effect on the surgical resection rate.

The followings are the main purposes of using neoadjuvant immunotherapy for NSCLC: to decrease the size of primary tumor and to narrow the extent of involved lymph nodes, thereby decreasing tumor staging, reducing surgical trauma, and increasing the likelihood and safety of surgical resection [36]. In addition, neoadjuvant therapy may reduce or eliminate tumor cells that remain after surgery, thereby reducing the risk of postoperative recurrence and metastasis and subsequently improving overall survival [37]. Several retrospective studies have shown that pathologic response after neoadjuvant therapy is strongly associated with the odds of surgical recurrence and overall survival [38, 39, 40, 41]. In addition, compared with adjuvant therapy, neoadjuvant therapy can target tumor antigens present in the pre-resection tumor, thus provoking an immune response of antitumor T cells to better act on tumor micro-metastatic lesions [42].

The internal and external environments where tumor cells reside, also including endothelial, stromal, and immune cells, termed the tumor microenvironment (TME) [43]. Recently, it has been shown that the immune cell composition in TME changes dramatically even in the early stages of the tumor, which may synergistically promote the immunosuppressive microenvironment and cascaded promote tumor progression [44]. Therefore, immunotherapy is important for comprehensive treatment of patients with tumors at early stage, and neoadjuvant immunotherapy with ICIs administered before surgery in patients with NSCLC at early stage might induce a durable antitumor T-cell immune response, which in turn is more effective in preventing tumor recurrence. The reason for these may be related to the following mechanisms: (I) neoadjuvant immunotherapy could increase the number of activated tumor specific CD8^+^ T cells, and new tumor antigens could be released for presentation to tumor-specific effector T cells that can directly killing the tumor on both the primary and metastatic sites, as well as in the circulatory system; (II) activated T cells could target metastatic tumors in blood vessels and lymphatic vessels, triggering a series of specific antitumor immune responses; (III) compared with postoperative adjuvant immunotherapy, which can act on the undisrupted peritumoral lymphatic system, the presence of broader tumor neoantigens may enhance immune recognition, generating more robust early antitumor immune responses and immunological memory [45].

Currently, many perioperative treatments, such as radiotherapy, chemotherapy, immunotherapy, and targeted therapy, are being explored to decrease the risk of postoperative recurrence and gain long-term survival benefit in resectable NSCLC. The development of neoadjuvant and adjuvant therapies can effectively improve the effective rate of surgery, increase the proportion of radical resection, reduce postoperative complications, and enable surgical patients to obtain greater benefits. ICIs are currently a research hotspot for the perioperative treatment of tumors, starting to be used as maintenance therapy after the failure of multiple lines of treatment for advanced lung cancer, and now gradually applied to early-stage NSCLC [46]. With the large number of clinical trials and the publication of results, many important breakthroughs in the evaluation of efficacy of ICIs, predictive biomarkers, and their safety profiles have been affirmed. These findings indicated that neoadjuvant immunotherapy is one the most promising treatment strategies for the treatment of resectable NSCLC [47]. The ongoing clinical trials include (I) small phase II chemoimmunotherapy studies; (II) multiple small phase II neoadjuvant immunotherapy studies; (III) phase III adjuvant chemoimmunotherapy studies; and (IV) phase III neoadjuvant chemotherapy plus immunotherapy followed by different lengths of postoperative adjuvant immunotherapy [48].

Some limitations exist in the present meta-analysis. (I) The sample size is small, and most of the clinical trials are still ongoing with only partial initial results published; and (II) there is a small proportion of RCTs in the published studies, and the biased results are obvious, which affects the science and rigors of the study. Nevertheless, our meta-analysis provided evidence-based medical insights, demonstrated the safety and efficacy of neoadjuvant immunotherapy for resectable NSCLC, and provided guidance for clinical practical application and future clinical trials.

## 5. Future Perspectives

Immunotherapy holds great promise, but it also faces challenges. Firstly, neoadjuvant and adjuvant immunotherapy have a significant survival benefit compared with chemotherapy, but the use of immunotherapy, especially neoadjuvant immunotherapy, may cause autoimmune diseases, which may lead to delayed surgical time and even inability to tolerate surgery in patients [49, 50]. In addition, the premature use of immunotherapy in lung cancer at early stage may promote the resistance of immunotherapy and add difficulty to subsequent comprehensive antitumor therapy [51]. In addition, whether neoadjuvant immunotherapy will aggravate the incidence of complications such as bleeding during surgery, increase the difficulty of surgery, and prolong the duration of chest drainage needs to be further evaluated [52]. Finally, patients should be screened for indications using validated molecular markers, but so far, it is controversial to use PD-L1, TMB, or, more recently, liquid biopsies (ctDNA, peripheral blood T cells, etc.), with insufficient evidence of a direct association with MPR or OS, Screening suitable patients by these markers remains [53, 54]. In future studies, it will be important to find a comprehensive index that reflects both tumor response and immune response, and only then, we can truly monitor the effect of immunotherapy in real time.

## 6. Conclusion

In summary, our study showed that neoadjuvant immunotherapy is more effective than neoadjuvant chemotherapy, and its safety has also been verified. Subgroup analysis showed that there was no significant difference in the efficacy between different ICIs. Neoadjuvant immunotherapy plus chemotherapy did not significantly increase the occurrence of TRAEs and did not cause any delay in surgery compared with neoadjuvant chemotherapy alone.

## Figures and Tables

**Table 1:** Summary of characteristics of studies on neoadjuvant immunotherapy in resectable NSCLC.

Clinical trail	NCT number	Study type	Type of article	Study phase	Main inclusion criteria	ICI	Sample size	Proportion of gender	Median age	MPR	CPR	Incidence of TRAE	Grade 3 or higher TRAEs	Surgical resection rate	Incidence of surgical complications	Surgical delay rate	First author	Published year	Ref
CheckMate 816	NCT02998528	RCT	Conference abstract	III	Resectable stage I-IIIA NSCLC	Nivolumab	21	NR	NR	45% (9/20)	15% (3/20)	NR	NR	95.2% (20/21)	NR	0	Felip E	2018	[8]
NEOSTAR	NCT03158129	Cohort study	Article	II	Resectable stage I-IIIA (single N2) NSCLC	Nivolumab ± ipilimumab	44	16 (36%) women and 28 (64%) men	66 (43–83) y	29.4% (10/34)	17.6% (6/34)	NR	12.2% (5/44)	82.9% (34/41)	29.4% (10/34)	NR	Cascone	2021	[9]
NR	NCT02716038	Cohort study	Article	II	Resectable stage IB-IIIA NSCLC	Atezolizumab + chemo (nab-paclitaxel and carboplatin)	30	15 (50%) women and 15 (50%) men	67 (62–74) y	56.7% (17/30)	33.3% (10/30)	NR	NR	86.7% (26/30)	NR	0%	Shu CA	2020	[10]
NR	ChiCTR-OIC-17,013,726	Cohort study	Article	Ib	Resectable stage IA-IIIB NSCLC	Sintilimab	40	8 (20%) women and 32 (80%) men	61.5 (48−70) y	40.5% (15/37)	16.2% (6/37)	52.5% (21/40)	10% (4/40)	92.5% (37/40)	10% (4/40)	5% (2/40)	Gao	2020	[11]
NR	NCT02259621	Cohort study	Article	I	Stage I-IIIA biopsy-proven NSCLC	Nivolumab	22	11 (52%) women and 10 (48%) men	67 (55–84) y	45% (9/20)	10% (2/20)	23% (5/22)	4.5% (1/22)	91.0% (20/22)	50% (10/20)	0%	Bott MJ	2019	[12]
NR	NCT02259621	Cohort study	Article	NR	Resectable stage I-IIIA NSCLC	Nivolumab	21	11 (52%) women and 10 (48%) men	67 (55–84) y	45% (9/20)	15% (3/20)	23% (5/21)	4.7% (1/21)	95.2% (20/21)	NR	0%	P.M. Forde	2018	[13]
NR	NCT02994576	Cohort study	Conference abstract	II	Stage IA (≥2 cm)-IIIA non-N2, resectable, and untreated NSCLC	Atezolizumab	30	15 (50%) women and 15 (50%) men	64 y	13.3% (4/30)	0%	3.3% (1/30)	0%	100% (30/30)	NR	0%	Besse	2020	[14]
NADIM	NCT03081689	Cohort study	Article	II	Resectable stage IIIA NSCLC	Nivolumab + chemo (paclitaxel and carboplatin)	46	12 (26%) women and 34 (74%) men	63 (58–70) y	83% (34/41)	63% (26/41)	93% (43/46)	30% (14/46)	89% (41/46)	29% (12/41)	NR	Provencio	2020	[15]
NR	NCT02259621	Cohort study	Article	NR	Resectable stage IB –IIIA NSCLC	nivolumab + ipilimumab	9	2 (22%) women and 7 (78%) men	61.7 (48–78) y	33% (2/6)	33% (2/6)	66% (6/9)	33% (3/9)	66.7% (6/9)	NR	0%	Reuss JE	2020	[16]
NR	NCT02818920	Cohort study	Article	II	Untreated clinical stage IB-IIIA NSCLC	Pembrolizumab	30	14 (47%) women and 16 (53%) men	72 (47–81) y	28% (7/25)	12% (3/25)	NR	3.3% (1/30)	83.3% (25/30)	48% (12/25)	4% (1/25)	Tong, B. C.	2021	[17]
NEOMUN	NCT03197467	Cohort study	Article	II	Resectable NSCLC stage II/IIIA	Pembrolizumab	15	8 women, 7 men	59.8 (40–83) y	27% (4/15)	13% (2/15)	33% (5/15)	13% (2/15)	100% (15/15)	7% (1/15)	7% (1/15)	Eichhorn, F.	2021	[18]
NR	NR	Cohort study	Article	NR	Untreated, surgically resectable stage IIB–IIIB LUSC	Pembrolizumab + chemo (nab-paclitaxel and carboplatin)	37	2 (5.4%) women and 35 (94.6%) men	62.8 (38–76) y	64.9% (24/37)	45.9% (17/37)	70.3% (26/37)	10.8% (4/37)	100% (37/37)	NR	0%	Shen, D.	2021	[19]
NR	NR	Cohort study	Article	NR	Stages IIA–IIIB NSCLC	PD-1 + chemo	23	1 (4.3%) woman and 22 (95.7%) men	61.83 y	50% (10/20)	30% (6/20)	26% (6/23)	4% (1/23)	87% (20/23)	25% (5/20)	0%	Duan, Ht	2021	[20]
NR	NR	Cohort study	Article	NR	Stage IIIA NSCLC	PD-1 + chemo (nab-paclitaxel and carboplatin)	72	6 (8.3%) women and 66 (91.7%) men	62.2 (42–76) y	65.2% (47/72)	29.1% (21/72)	NR	6.9% (5/72)	100% (72/72)	NR	0	Wang Jf	2021	[21]
NR	NCT02904954	RCT	Article	II	Resectable stage I-IIIA NSCLC	Durvalumab ± RT (8 Gy × 3)	60	29 (48%) women and 31 (52%) men	71.5 (64–75) y	34.6% (18/52)	15.3% (8/52)	NR	18.3% (11/60)	87% (52/60)	38.5% (20/52)	3.8% (2/52)	Altorki NK	2021	[22]
SAKK 16/14	NCT02572843	Cohort study	Article	II	Locally advanced T1-3N2M0, stage IIIA (N2) NSCLC	Durvalumab + chemo (docetaxel and cisplatin)	67	35 (52%) men and 32 (48%) women	61 (41–74) y	62% (34/55)	18% (10/55)	NR	88% (59/67)	82% (55/67)	NR	NR	Rothschild	2021	[23]
LCMC3	NCT02927301	Cohort study	Conference abstract	II	Stages IB-selected IIIB resectable NSCLC	Atezolizumab	101	54 (53.5%) women and 47 (46.5%) men	64 y	18% (15/82)	4.9% (4/82)	5.9% (6/101)	NR	98% (90/101)	27.8% (25/90)	NR	Kwiatkowski DJ	2019	[24]
MK3475−223	NCT02938624	Cohort study	Conference abstract	I	Stage I-II NSCLC	Pembrolizumab	10	NR	NR	40% (4/10)	NR	NR	NR	100% (10/10)	0% (0/10)	0%	Bar J	2019	[25]

## Data Availability

The datasets presented in this study are available from the corresponding author upon request.
